# Interplay Between TGFβ1 Signaling and Cancer-Testis Antigen MAGEB2: A New Thorn in Cancer’s Side?

**DOI:** 10.3390/ijms26062448

**Published:** 2025-03-09

**Authors:** Ashley Colemon, Carlan V. Romney, Angelle D. Jones, Clarke Bagsby, Richala Jackson, Saumya Ramanathan

**Affiliations:** 1 Department of Life and Physical Sciences, Fisk University, Nashville, TN 37208, USA; 2 Fisk-Vanderbilt Master’s-to-Ph.D. Bridge Program, Nashville, TN 37208, USA

**Keywords:** cancer-testis antigens, cancer, epigenetics, MAGEB2, cell proliferation

## Abstract

The Melanoma Antigen Gene (MAGE) family of proteins is the largest family of cancer-testis antigens (CTAs) and shares a MAGE homology domain (MHD). MAGE proteins are divided into Type I and Type II MAGEs depending on their chromosomal location and expression patterns. Type I MAGEs are true CTAs. MAGEB2 is a Type I MAGE, belonging to the MAGEB subfamily, and unlike some MAGE proteins, has not been found to bind to and enhance E3 ligase activity. MAGEB2 has been discovered to be an RNA-binding protein that serves to protect spermatogonial cells in the testis from extraneous stressors. We have discovered that MAGEB2 is necessary and sufficient for the proliferation of cells and is expressed by the differential DNA methylation of its gene promoter. Furthermore, we identified JunD as the transcription factor that regulates MAGEB2 expression. When expressed, MAGEB2 suppresses transforming grown factor-β1 (TGFβ1) signaling by decreasing mRNA levels of Thrombospondin-1 (TSP-1). TSP-1 is an anti-angiogenic protein that activates TGFβ1. Restoring levels of TSP-1 or TGFβ1 results in the inability of MAGEB2 to drive proliferation, suggesting that MAGEB2-expressing tumors might be more susceptible to therapies that induce or activate TSP-1 or TGFβ1 signaling.

## 1. Introduction

Cancer-testis antigens (CTAs) are a group of tumor-specific proteins that are physiologically restricted to expression in the testis and then aberrantly re-expressed in a wide variety of cancers [1]. The Melanoma Antigen Gene (MAGE) family is the largest family of CTAs, with ~60 human genes, including pseudogenes [2,3]. MAGEs are divided into Type I and Type II MAGEs based on their expression pattern and chromosomal location. Type I MAGE genes are expressed in the testis and a wide variety of cancers, adhering to the definition of CTAs, and are all located on the X-chromosome. Type II MAGEs, on the other hand, are expressed ubiquitously in all somatic tissues, and while some are on the X-chromosome, some are located on autosomes as well [4]. Of the Type I MAGEs, the MAGEA subfamily is the most well studied, and some MAGEA proteins bind to and enhance the activity of E3-Ubiquitin ligases with important biochemical implications [5,6]. For example, MAGEA3/A6 binds to TRIM28 and enhances the ubiquitination and degradation of the tumor suppressor AMPK [7]. Similarly, the activation of the MAGEA11-HUWE1 complex results in the enhanced ubiquitination of PCF1, which causes the alternative polyadenylation of several mRNA transcripts that regulate cell proliferation [8].

Type I MAGEs have garnered significant interest in the past few decades due to their potential use as cancer vaccines [9,10]. On the flip side, they are also of interest because several MAGE proteins contribute to pro-proliferative phenotypes and are tumor-specific in expression [6,8], making them potential cancer drug targets. In addition, expression of certain MAGE proteins has been discovered to be a harbinger of outcomes of cancer therapies [11]. The epigenetic regulation of gene expression has long been accepted as pivotal in tumor formation [12,13,14,15]. Therefore, the use of epigenome modifiers as a cancer therapy, either alone or in combination with other drugs, is beginning to gain traction [16,17]. The classic paradox regarding cancer-testis antigens and, specifically, the MAGE proteins, is that epigenome modifiers might cause the expression of these genes, which then confer survival and proliferative phenotypes on cells. As cancer therapeutics moves towards personalized medicine [18,19,20,21], knowing the expression status of the MAGE genes and what phenotype they might confer, if any, to cells will certainly aid in making the best decisions.

**Figure 1:**
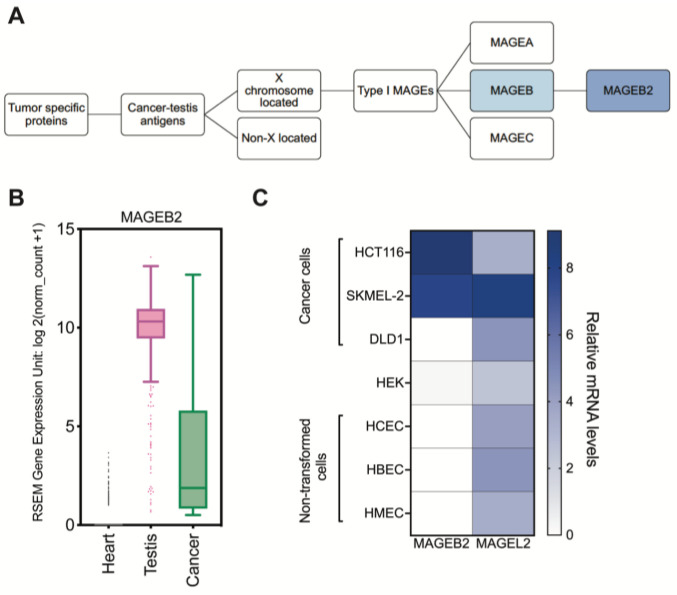
Introduction to MAGEB2 and its relationship with the MAGE family of proteins. (**A**) Schematic illustrating the focus of this paper. Melanoma Antigen Genes are divided into Type I and Type II based on their expression pattern and their chromosomal location. Type I MAGEs are bona fide cancer-testis antigens and are located on the X-chromosome, whereas Type II MAGEs display ubiquitous expression and are not restricted to the X chromosome. MAGEB2 is a Type I MAGE belonging to the MAGEB subfamily and is the focus of this paper. (**B**) MAGEB2 is a true cancer-testis antigen. The mRNA expression of *MAGEB2* from TCGA PAN-Cancer and GTEX datasets in heart (black), testis (pink), and cancer (green). (**C**) The RT-qPCR expression of *MAGEB2* and *MAGEL2* genes in primary, immortalized human mammary (HMEC), bronchial (HBEC) and colonic epithelial cells (HCEC), HEK cells, and in patient-derived cancer cell lines DLD-1, HCT116 (colon cancer), and SKMEL-2 (melanoma). *MAGEL2* is a ubiquitously expressed Type II MAGE.

The *MAGEB* subfamily of the Type I MAGEs consists of nine protein-encoding genes in humans. None of the MAGEB proteins have been discovered to bind to E3-Ubiquitin ligases. While all MAGE proteins share a common MAGE Homology Domain (MHD) [5], the sequence identity between a MAGEB protein and a MAGEA protein is between 40 and 60% [3]. MAGEB proteins are not well studied, and their biochemical functions are mostly unknown. Similarly, mechanisms that regulate expression of the *MAGEB* subfamily of genes are not well understood. This study focuses on MAGEB2, which has been recently discovered as an RNA-binding protein that regulates stress response in spermatogonial cells [22,23] (Figure 1A). MAGEB2 plays a protective role against extraneous stressors and, therefore, we have tested the hypothesis that the expression of MAGEB2 is regulated by epigenetic mechanisms and, when expressed, MAGEB2 provides a proliferative and survival advantage to cells.

## 2. Results

### 2.1. MAGEB2 Is a True Cancer-Testis Antigen

In order to determine whether *MAGEB2* strictly adhered to the expression pattern of a cancer-testis antigen, we used the UCSC Xena functional genomics browser to analyze the mRNA expression of *MAGEB2* from RNA-sequencing datasets from the TCGA (PAN-CANCER) and GTEx dataset for heart and testis samples [24]. Our analysis of the data confirms that *MAGEB2* is highly expressed in the testis and variably expressed in pan-cancer tissues, and we saw none-to-minimal expression in the heart tissues (Figure 1B). To confirm whether this expression profile translates into cell culture models, we next performed an RT-qPCR analysis on non-transformed cells and cancer cell lines. We measured the relative mRNA levels of *MAGEB2* and *MAGEL2* in normal non-transformed cell lines from three different tissue origins: breast (human mammary epithelial cells, HMEC), lung (human bronchial epithelial cells, HBEC), and colon (human colonic epithelial cells, HCEC). *MAGEL2* is a Type II MAGE that is expressed ubiquitously in all somatic tissues [3]. We detected no expression of *MAGEB2* in the non-transformed cell lines (Figure 1C). HEK 293 cells have a low basal expression of *MAGEB2.* Patient-derived cancer cell lines such as HCT116 (colon cancer) and SK-MEL-2 (melanoma) displayed a 6–8-fold higher expression of *MAGEB2* (Figure 1C). Interestingly, DLD-1 (colon cancer) did not express *MAGEB2. MAGEL2*, a Type II MAGE, on the other hand, is ubiquitously expressed in non-transformed and cancer cells. Taken together, these data indicate that *MAGEB2* is not expressed in any normal tissue or cell lines, but is highly, and variably, expressed in cancer cells and, therefore, is a true cancer-testis antigen.

### 2.2. DNA Methylation Regulates Expression of MAGEB2

**Figure 2:**
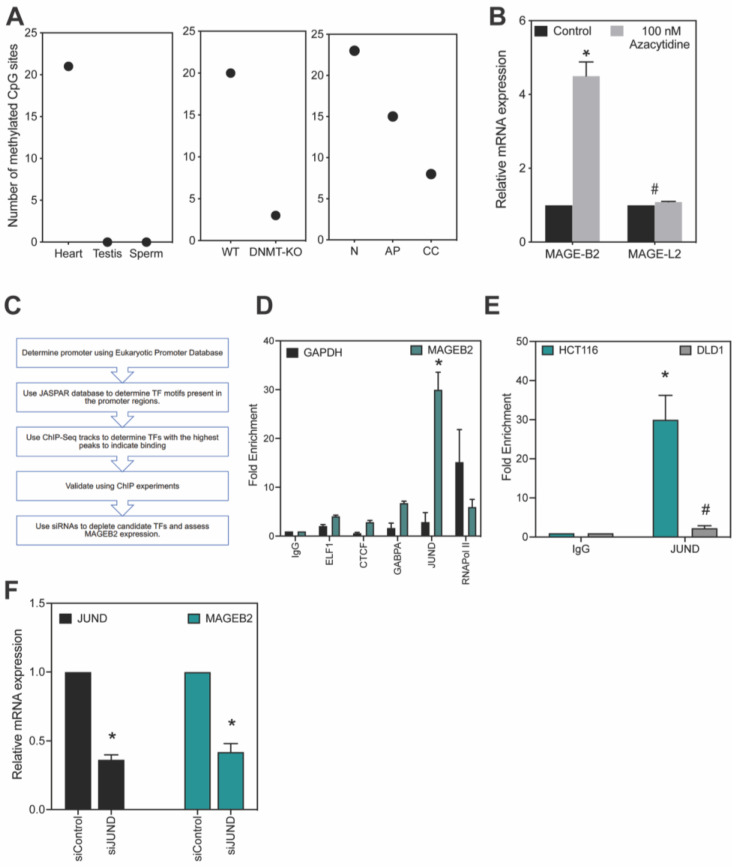
*MAGEB2* expression is regulated by hypomethylation and transcription factor JunD. (**A**) The number of fully methylated CpG sites 1000 bp upstream of the transcription start site of the *MAGEB2* gene were counted using methylome datasets in the UCSC genome browser in the indicated tissue: (H) heart, (T) testis, and (S) sperm (left panel). The number of fully methylated CpG sites 1000 bp upstream of the transcription start site of the *MAGEB2* gene were counted using two methylome datasets in the UCSC genome browser in the HCT116 wild-type cell line (WT) and HCT116, where DNMT1 and DNMT3b (DNA methyl transferases) were depleted (DKO) (middle panel). The number of fully methylated CpG sites 1000 bp upstream of the transcription start site of the *MAGEB2* gene were counted using methylome datasets in the UCSC genome browser in the indicated tissue: (N) normal colon, (AP) adenomatous polyp, and (CC) colon cancer tissue (right panel). (**B**) The mRNA expression of *MAGEB2* and *MAGEL2* was determined by RT-qPCR in HEK cells treated with DMSO (black) or 100 nM azacytidine (grey). Data shown are mean ± S.D. from n = 3 experiments. *p* values were determined from Students’ *t*-test, * indicates *p*-value < 0.001, and # is not significant (NS). (**C**) Flowchart illustrating our methods in determining the transcription factor that regulates the expression of *MAGEB2*. (**D**) Chromatin immuno-precipitation (ChIP) was performed in HCT116 cells using antibodies against each respective transcription factor. The fold enrichment of each transcription factor on either the *GAPDH* or *MAGEB2* gene promoter was analyzed using qPCR. RNA polymerase II was used as an internal positive control. Data shown are mean ± S.D. from n = 3 experiments. *p* values were determined from Student’s *t*-test and * indicates *p*-value < 0.01. (**E**) Chromatin immuno-precipitation (ChIP) was performed in HCT116 cells and DLD-1 cells with a control IgG or JunD antibody. The fold enrichment of JunD on the *MAGEB2* gene promoter was analyzed using qPCR. Data shown are mean ± S.D. from n = 3 experiments. *p* values were determined from Student’s *t*-test, * indicates *p*-value < 0.01, and # is not significant (NS). (**F**) The siRNA-mediated knockdown of JunD was performed in HCT116 cells, and the expression of JunD and MAGEB2 were determined by RT-qPCR. Data shown are mean ± S.D. from n = 3 experiments. *p* values were determined from Student’s *t*-test and * indicates *p*-value < 0.01.

The expression of several cancer-testis antigens, including *MAGE-A* genes, *XAGE3*, and *NY-ESO-1*, is regulated by DNA methylation [6,25,26]. Given this and the fact that the cancer cell lines exhibited heterogeneity in the expression of *MAGEB2* (Figure 1C)*,* we sought to determine whether DNA methylation played a role in the expression of *MAGEB2*. We first performed a bioinformatics analysis using the UCSC genome browser and determined the number of methylated CpG sites 1000 bp upstream of the transcription start sites of *MAGEB2* in tissues where *MAGEB2* expression is high (testis and sperm) versus low or non-existent (heart). Consistent with our hypothesis, there is a distinct pattern of hypomethylation in the testis and sperm, where the *MAGEB2* gene is expressed (Figure 2A, left panel). We then determined the number of methylated CpG sites in patient-derived cancer line HCT116 (WT) versus HCT116 cells with a double knock-out for DNA-methyltransferases (DNMTs) *DNMT1* and *DNMT3b.* As shown in Figure 2A (middle panel), in two independent studies [27,28], the number of methylated cytosines decreases in the *MAGBE2* promoter region when DNA-methyltransferases (DNMT1 and DNMT3b) are depleted. We then analyzed the methylation status of *MAGEB2* promoter in normal colon, adenomatous polyp, and colon cancer tissue [29]. There was a concomitant decrease in the methylation of the *MAGEB2* promoter in colon cancer tissue when compared to normal colon mucosa or adenomatous polyp (Figure 2A, right panel).

We used a DNMT inhibitor 5-azacytidine [30] to ascertain whether prolonged and low-dose treatment could elicit *MAGEB2* expression. The expression of *MAGEB2* increased upon treatment of HEK cells with 100 nM 5-azacytidine compared to the DMSO control, and, more evidently, the expression of *MAGEL2*, a type II MAGE, was not altered (Figure 2B). Therefore, our data indicate that DNA methylation regulates the expression of *MAGEB2*.

### 2.3. JunD Is the Transcription Factor That Regulates the Expression of MAGEB2

While epigenetic mechanisms can act as switches to regulate expression of genes, the recruitment of transcription factors is still necessary for transcription. Therefore, we designed a workflow to determine the transcription factor(s) (TFs) required for *MAGEB2* transcription (Figure 2C). Briefly, using a bioinformatics approach, we first determined the predicted *MAGEB2* promoter region, followed by determining the consensus TF-binding motifs in this region. Then, we analyzed the UCSC Genome Chromatin Immuno-precipitation Sequencing (ChiP Seq) data to determine which TFs were bound to *MAGEB2* promoters in tissues/cells that expressed *MAGEB2*. Finally, we followed this up with experiments by performing chromatin immuno-precipitation with antibodies to candidate TFs and determined binding to the *MAGEB2* promoter. We then deleted candidate TFs and analyzed the expression of *MAGEB2* (Figure 2C).

Bioinformatics analysis revealed that four putative TFs were bound to the *MAGEB2* promoter: GABP-a, ELF1, CTCF, and JunD. Our data from the ChIP assay show that JunD was ~10-fold more enriched on the *MAGEB2* promoter than on the GAPDH promoter in HCT116 cells, whereas other candidate TFs were <2-fold enriched on the *MAGEB2* promoter compared to GAPDH (Figure 2D). To confirm that JunD was indeed the transcription factor regulating the expression of *MAGBE2,* we then performed the ChIP assay in DLD-1 cells, where *MAGEB2* is not expressed (Figure 1C). In DLD-1 cells, JunD was not enriched in the region of the *MAGEB2* promoter (Figure 2E), thus indicating that the binding of JunD to the *MAGEB2* promoter has a functional consequence. Finally, the depletion of JunD expression with siRNAs resulted in decrease in *MAGEB2* expression by more than 50% compared to control siRNA (Figure 2F). Taken together, our data indicate that, while the hypomethylation of the CpG islands near the *MAGEB2* promoter regulates the expression of the MAGEB2 gene, JunD is the transcription factor that causes the expression of MAGEB2 in cells.

### 2.4. MAGEB2 Is Both Necessary and Sufficient for Cellular Proliferation

**Figure 3:**
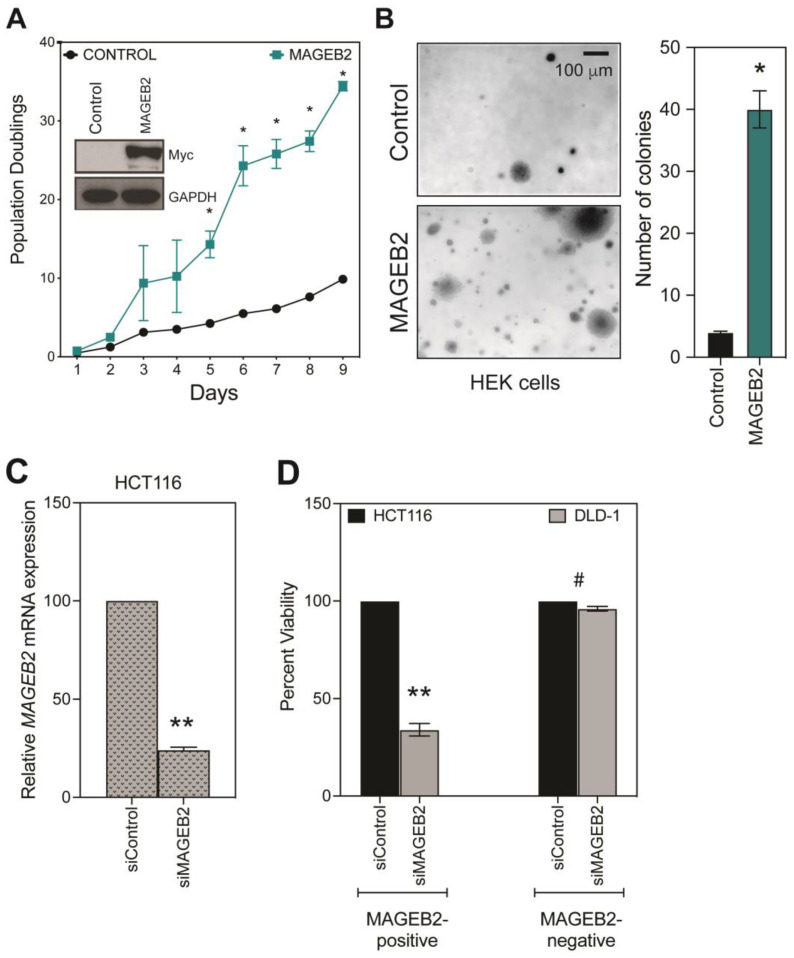
*MAGEB2* is both sufficient and necessary for cell proliferation. (**A**) HEK cells expressing vector control or myc-tagged MAGEB2 were lysed, and samples were resolved on SDS-PAGE followed by Western blotting with anti-myc antibody to verify the expression of myc-MAGEB2 (inset). The population doubling of HEK cells expressing vector control or myc-tagged MAGEB2 over a period of 9 days was determined by counting cells every 24 h. Data shown are mean ± SEM from n = 3 experiments. *p* values were determined from Students’ *t*-test and * indicates *p*-value < 0.01. (**B**) HEK cells expressing vector control or MAGEB2 were plated in soft agar colony formation assay and colonies were imaged and counted on day 15. Data shown are mean ± S.D. from n = 3 experiments. *p* values were determined from Students’ *t*-test and * indicates *p*-value < 0.01. (**C**) HCT116 cells were treated with siControl or siMAGEB2 for a period of 48 h, and, at 72 h, they were harvested for RT-qPCR to determine the efficacy of MAGEB2 knockdown. Data shown are mean ± S.D. from n = 3 experiments. *p* values were determined from Students’ *t*-test and ** indicates *p*-value < 0.001. (**D**) HCT116 cells (MAGEB2 positive) and DLD-1 cells (MAGEB2 negative) were treated with siControl or siMAGEB2 for a period of 48 h, and cell viability was measured at 72 h post treatment using the end point Cell-Titer Glo assay. The luminescence of cells treated with siControl was set to 100% viability. Data shown are mean ± S.D. from n = 3 experiments. *p* values were determined from Students’ *t*-test, ** indicates *p*-value < 0.001, and # is not significant (NS).

To determine whether the aberrant re-expression of *MAGEB2* in cancer has functional consequences, or whether it is caused due to the generalized and rampant de-regulated expression of many genes, we performed gain- and loss-of-function studies. We first expressed *MAGEB2* in HEK cells (Figure 3A, inset). We then assayed for cell proliferation rates over 9 days. Cells expressing MAGEB2 proliferated faster than control cells (Figure 3A). Similarly, MAGEB2 expression leads to the formation of an ~8-fold increase in the number of colonies compared to cells expressing the vector control (Figure 3B). Our data indicate that MAGEB2 expression is sufficient to drive increased cell proliferation. Next, we depleted MAGEB2 from HCT116 cells, where MAGEB2 is highly expressed (Figure 1C), and used DLD-1 cells as our negative control. The depletion of MAGEB2 with siRNAs for 72 h results in a >50% decrease in the cell viability of patient-derived HCT116 cancer cells. DLD-1 cells displayed no decrease in viability, indicating that our treatment conditions are not toxic to the cells (Figure 3D). These data indicate that MAGEB2 expression is necessary for the survival of cancer cells. Taken together, our data indicate that MAGEB2 expression is both sufficient and necessary for driving cellular proliferation.

### 2.5. MAGEB2 Expression Leads to the Suppression of TGFβ1 Signaling

**Figure 4:**
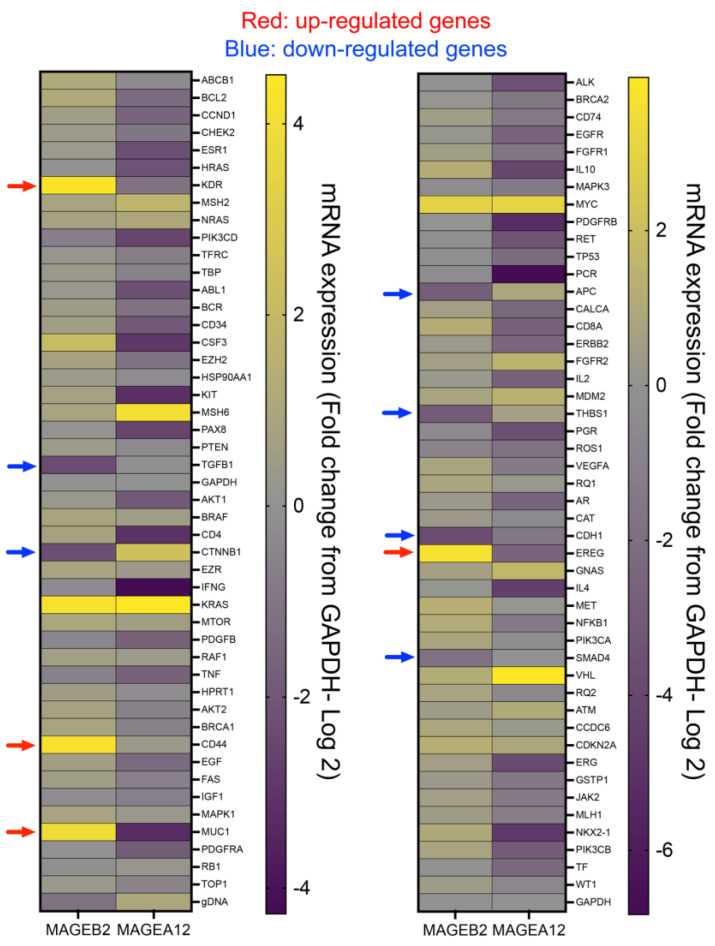
Expression of MAGEB2 results in modulation of TGFβ1 signaling. Heatmap showing changes in expression of 96 cancer-associated genes (including negative and positive controls) upon expression of either MAGEB2 or MAGEA12, as determined by RT-qPCR using Bio-Rad gene expression array. Red arrows indicate genes that were specifically up-regulated upon expression of MAGEB2, while blue arrows indicate those that were specifically down-regulated upon expression of MAGEB2.

**Figure 5:**
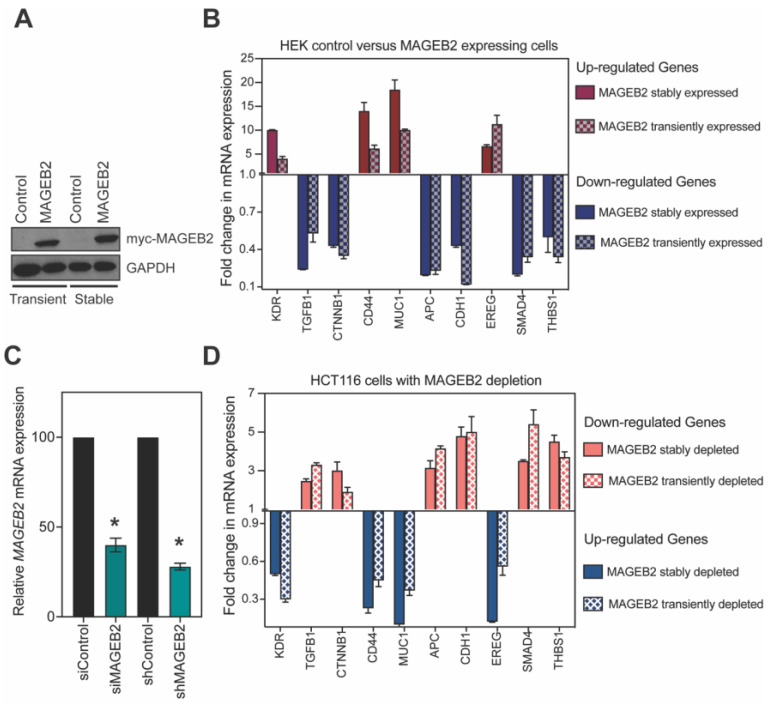
Expression of MAGEB2 results in down-regulation of TGFβ1 signaling. (**A**) HEK cells were either transiently transfected with vector control or MAGEB2-expressing plasmid and stably transduced with lentiviral particles expressing vector control of MAGEB2. Lysates were resolved on SDS-PAGE, followed by Western blotting with anti-myc antibody for MAGEB2 and GAPDH for loading control. (**B**) mRNA expression of the 10 genes that were modulated in the Bio-Rad RT-qPCR array were determined in HEK cells transiently or stably expressing MAGEB2. Data shown are mean ± S.D. from n = 3 experiments. (**C**) HCT116 cells treated with siControl or siMAGEB2 for 72 h, and those that were transduced and stably selected for expression of shControl or shMAGEB2 plasmids were harvested, and MAGEB2 mRNA levels were measured by RT-qPCR. Data shown are mean ± S.D. from n = 3 experiments. *p* values were determined from Students’ *t*-test and * indicates *p*-value < 0.001. (**D**) HCT116 cells depleted of MAGEB2 expression either transiently (siRNA) or stably (shRNA) were harvested. mRNA expression of the 10 genes that were modulated in the Bio-Rad RT-qPCR array were determined by RT-qPCR. Data shown are mean ± S.D. from n = 3 experiments.

To determine how MAGEB2 expression leads to increased proliferation (Figure 3), we performed gene expression analysis on control and MAGEB2-expressing cells using RT-qPCR arrays. We performed the experiment in replicates with control cells, MAGEB2-expressing cells, and MAGEA12-expressing cells. We used MAGEA12 as an additional “negative” control because MAGEA12 is a cancer-testis antigen and the expression of MAGEA12 causes increases in cell proliferation [6]; furthermore, MAGEA12 shares only 39% sequence identity with MAGEB2. This strategy would enable us to detect those genomic changes that would be caused by the expression of MAGEB2 specifically, and not just any CTA that causes an increase in proliferation. *KDR*, *CD44*, *MUC1*, and *EREG* were the top four genes to be up-regulated, specifically in *MAGEB2*-expressing cells. *TGFb1*, *CTNNB1*, *APC*, *CDH1*, *SMAD4*, and *THBS1* were the top six genes to be down-regulated, specifically, in *MAGEB2*-expressing cells (Figure 4). We then validated whether these gene expression changes were *MAGEB2*-dependent (Figure 5B). For this, we used cell lines where MAGEB2 was stably expressed, and cells where we transiently expressed MAGEB2 (Figure 5A). We depleted MAGEB2 from HCT116 cells transiently using siRNAs and stably using shRNA and measured the levels of the same 10 genes (Figure 5C,D). The depletion of MAGEB2 resulted in the reversal of gene expression changes that occur upon expressing MAGEB2. Further analysis of our data, using GSEA [31,32], show that 5 of the 10 genes were associated with TGFβ1 signaling. Specifically, the overall effect of expressing MAGEB2 was a down-regulation in those genes that were up-regulated in response to TGFβ1. In other words, expressing MAGEB2 results in the downregulation of TGFβ1 signaling.

### 2.6. MAGEB2 Expression Results in Decreased Levels of Secreted TGFβ1 and TSP-1

**Figure 6:**
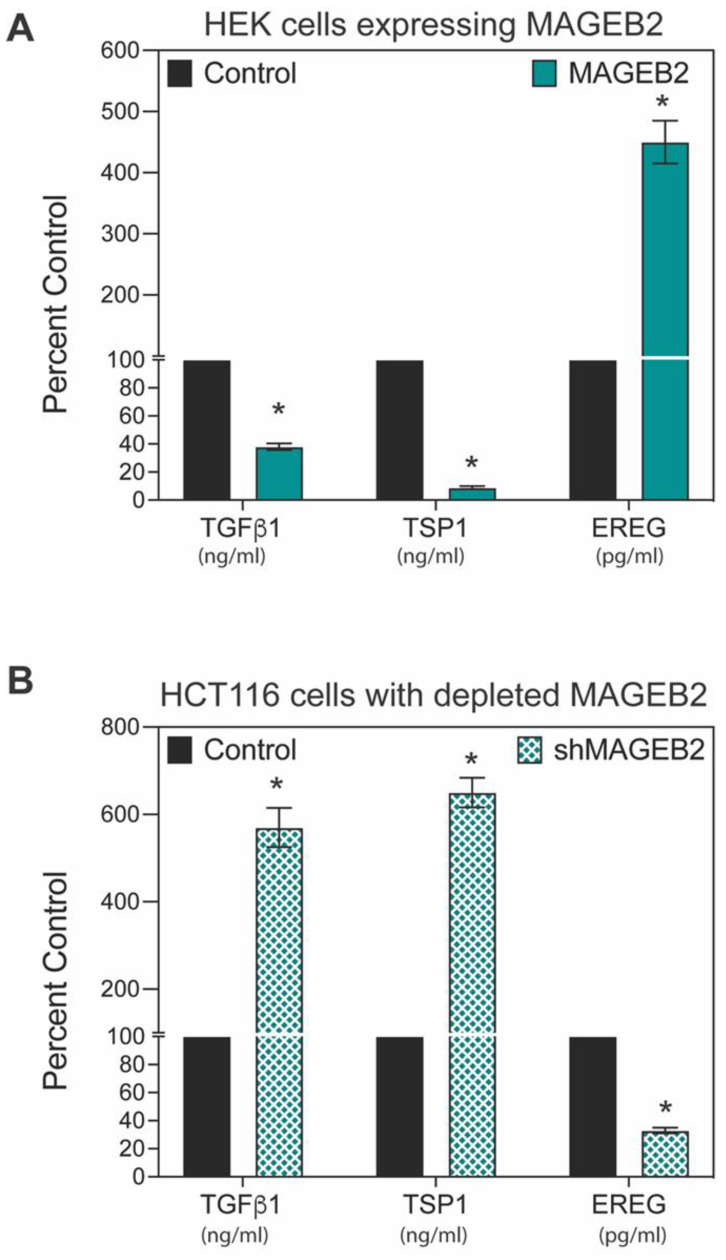
Expression of MAGEB2 results in decreased levels of secreted TSP1 and TGFβ1. (**A**) Conditioned media from HEK cells stably expressing vector control of MAGEB2 were harvested and Thrombospondin-1 (TSP-1), transforming growth factor (TGFβ1), and Epiregulin (EREG) levels were measured by ELISA. Data shown are mean ± S.D. from n = 3 experiments. *p* values were determined from Students’ *t*-test and * indicates *p*-value < 0.001. (**B**) Conditioned media from HCT116 stably expressing shControl or shMAGEB2 were harvested and Thrombospondin-1 (TSP-1), transforming growth factor (TGFβ1), and Epiregulin (EREG) levels were measured by ELISA. Data shown are mean ± S.D. from n = 3 experiments. *p* values were determined from Students’ *t*-test and * indicates *p*-value < 0.001.

In total, 3 of the 10 genes that were modulated by MAGEB2 expression are secreted factors, and to determine whether gene expression changes translated to protein levels, we measured the levels of EREG, TGFβ1, and TSP-1 in conditioned media secreted by control or MAGEB2-expressing cells using enzyme-linked immune assays (ELISA). Our data indicate that expressing MAGEB2 results in a >5-fold decrease in the levels of both TGFβ1 and TSP-1 (Thrombospondin-1) and a >5-fold increase in EREG (Epiregulin) (Figure 6A). In addition, the depletion of MAGEB2 from HCT116 cells results in the reversal of these changes. Specifically, when MAGEB2 is depleted, TGFβ1 and TSP-1 are increased, while EREG is decreased (Figure 6B).

### 2.7. Restoring TGFβ1 Levels Results in the Reversal of MAGEB2-Driven Anchorage-Independent Growth

**Figure 7:**
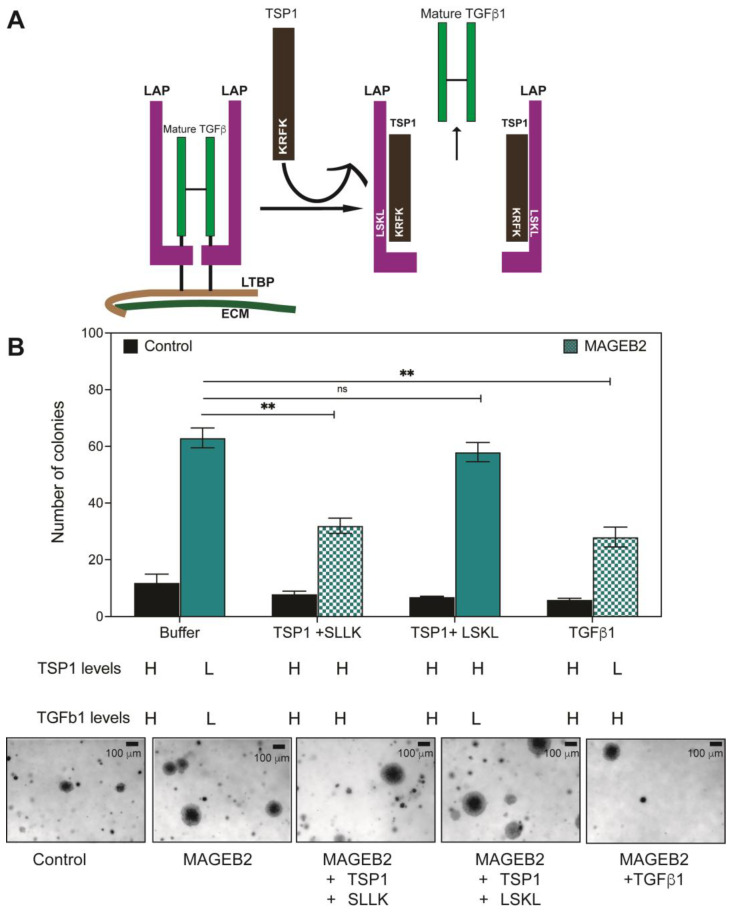
Restoring TGFβ1 levels results in rescue of MAGEB2-driven anchorage-independent growth. (**A**) Schematic illustrating how KRFK peptide within TSP-1 binds to LSKL peptide of latency-associated peptide (LAP) and causes release of active TGFβ1. (**B**) Single-cell suspensions of control (black bars) or MAGEB2-expressing (teal solid and patterned bars) cells were plated for soft agar colony formation assay. Media containing either buffer or TSP1 (1 μM) supplemented with either the SLLK (100 μM) or the LSKL (100 μM) peptide, or TGFβ1 (1 μM), were added every other day. Colonies were imaged and counted on day 15. Data shown are mean ± S.D. from n = 3 experiments. *p* values were determined from Students’ *t*-test, ** indicates *p*-value < 0.001, and ns indicates not significant. Predicted levels of TSP1 and TGFβ1 in each of the experimental conditions are indicated with H (high) or L (low).

Thrombospondin-1 (TSP-1) has long been known for its pleotropic effects in cancer, vascular diseases, and in immunity [33,34,35,36]. Thrombospondin-1 is a multi-domain protein secreted into the extra-cellular matrix [35,37]. One of the functions of TSP-1 is to bind to the latency-associated peptide (LAP) of the latent TGFb1 complex. This binding is mediated between the KRFK sequence in TSP-1 and the LSKL sequence in LAP, and results in a release of mature TGFb1 [38,39,40]. The exogenous LSKL peptide prevents the interaction of TSP-1 with LAP by competition and prevents the release and activation of TGFβ1 (Figure 7A). LSKL peptide is, therefore, an inhibitor of TSP-1, and has been explored in several clinical scenarios from cancer to hypertrophic scar formation and fibrosis [41,42,43,44,45,46]. When MAGEB2 is expressed in cells, we observed the decreased expression of TSP-1 and down-regulation of those genes that are typically up-regulated in response to TGFβ1 such as TGFb1, CTNNB1, CDH1, SMAD3, and APC (Figure 4). In addition, the expression of MAGEB2 depletes the levels of secreted TGFβ1 and TSP-1 (Figure 6). Given our data, we asked the question of whether restoring TSP-1 levels could attenuate MAGEB2-driven anchorage-independent growth. Indeed, when TSP-1 is supplemented in growth media for the duration of the colony formation assay, MAGEB2-expressing cells form 50% fewer colonies (Figure 7B). To determine whether the reason for this attenuation is related to the TGFβ1 activating function of TSP-1 or one of its other functions, we added either SLLK (control) or LSKL (TSP-1 antagonist) peptide to our colony formation assays. Finally, we added TGFβ1 to growth media. When TSP-1 and SLLK peptide are added, B2-driven increases in colony formation are rescued because TSP-1 is available to activate TGFβ1. When TSP-1 and LSKL peptide are added, MAGEB2 can still drive colony formation because TSP-1 can no longer activate TGFβ1 in the presence of the competitive antagonist LSKL. When TGFβ1 is added on its own, MAGEB2-driven colony formation is rescued. Therefore, our data indicate that active TGFβ1 is capable of decreasing MAGEB2-driven anchorage-independent growth (Figure 7B). More specifically, when the LSKL peptide antagonist is present, TSP-1 is unable to rescue anchorage-independent growth, suggesting that the TSP-1 activation of TGFβ1 is needed for rescuing MAGEB2-driven cell growth. We suspect that we do not see complete ablation (to control levels) of MAGEB2-driven colony formation in the presence of either TSP-1 or TGFβ1 because there might be additional signaling pathways that MAGEB2 is modulating to increase proliferation.

## 3. Discussion

Cancer-testis antigens have been of interest to scientists and clinicians due to their unique expression pattern; their expression is typically restricted to the germline, but is aberrantly expressed in a wide variety of cancers [1,2]. There are now more than 100 genes that fit this expression pattern, and the largest gene family of cancer-testis antigens are the Melanoma Antigen Genes (MAGEs) [47]. The biochemical function of a few MAGE proteins is to bind to and enhance the activity of their cognate E3 Ubiquitin ligases. The modification of substrate ubiquitination results in a cascade of signaling that leads to pleotropic effects such as altered vesicular trafficking (MAGEL2/TRIM27) [48], metabolic reprogramming and, therefore, the proliferative capacity of cells (MAGEA3/6/TRIM28) [7], and 3′ UTR shortening through alternative polyadenylation in cancer cells (MAGEA11/HUWE1) [8]. The MAGEB subfamily of proteins, however, have not been discovered to interact with E3 Ubiquitin ligases thus far, and the biochemical function of the MAGEB subfamily is largely a black box. Recently, MAGEB2 has been reported to be an RNA-binding protein that increases the stress tolerance of spermatogonial cells and cancer cells [22,23,49,50].

The alteration of homeostasis often signals the onset of carcinogenesis. The agents that alter homeostasis can be external or intrinsic stressors. For example, DNA damage, oxidative stress, unfolded protein response, and oxygen or nutrient deprivation are all linked to the onset of cancer [51,52] and, paradoxically, all must be bypassed for tumor survival. The connection between stressors and the epigenetic modulation of gene expression is also well documented during cell state changes [53,54]. In this complicated web of stress responses, we hypothesize that, during the early onset of cellular transformation, cells that are equipped to absorb the extraneous stressors have a proliferative advantage.

In this study, we determined whether CpG methylation, a well-known epigenetic modulator of gene expression, regulates MAGEB2 expression, and, furthermore, we discovered that JunD, a transcription factor known to play a protective role in the stress response [55], is recruited to the MAGEB2 promoter and drives the gene expression of MAGEB2. The expression of MAGEB2 increases cell proliferation and survival; the investigation of the pathways by which MAGEB2 achieves this revealed that MAGEB2 suppresses the expression of TSP-1/TGFβ1. Activating the TSP-1/TGFβ1 axis results in nullifying MAGEB2-driven cellular proliferation. This is an interesting finding, primarily due to the double-edged role that TGFβ1 plays in tumorigenesis. TGFβ1 is tumor-suppressive in early cancer development, but, in established tumors, it allows for tumor survival by creating immunosuppressive microenvironments, by increasing angiogenesis, and by regulating epithelial-to-mesenchymal transition [56,57,58,59]. This is in line with our hypothesis and data that MAGEB2 is expressed during the early onset of cancer via epigenetic mechanisms such as CpG methylation for the express purpose of conferring survival to cells. Based on our findings, treating MAGEB2-positive tumors with activators of TGFβ1 or TSP-1 signaling might be beneficial. This is not unprecedented; a study has found that MAGE-A3/6 expression predicts the resistance to CTLA-4 blockade therapy in melanoma patients [11]. Overall, we surmise that knowing the MAGE expression status of tumors is critical to determining the outcomes of therapies.

## 4. Materials and Methods

### 4.1. Cell Lines and Culture

Human colonic epithelial line (HCEC-CT) was cultured and maintained as described in [5,39]. Human bronchial epithelial cells (HBEC3-KT) were cultured and maintained at described in [40]. Human mammary epithelial cells were purchased from ATCC^TM^ (PCS600-010^TM^) and maintained in mammary epithelial cell medium supplemented with mammary epithelial cell growth kit (American Type Culture Collection ATCC, Manassas, VA, USA, PCS600-030^TM^ and PCS600-040^TM^). HEK293 cells were a kind gift from Dr. Shawn Goodwin at Meharry Medical College and were maintained in DMEM with 10% fetal bovine serum (FBS) and antibiotics. The 293 FT cells used for lentivirus generation were purchased from Thermo Fisher (R70007) and were cultured in DMEM with 10% FBS and antibiotics. HCT116 (ATCC, CCL-247), DLD-1 (ATCC, CCL-221), and SK-MEL2 cells were a kind gift from Dr. Ann Richmond at Vanderbilt University and were grown in DMEM with 10% FBS and antibiotics. All cells were grown at 37 °C with 5% CO_2_ unless otherwise indicated.

### 4.2. Antibodies

CTCF antibody (Cell Signaling, 2899) (Danvers, MA, USA), GABP-a (Santa Cruz Biotech, sc-28312) (Dallas, TX, USA), ELF1 (Santa Cruz Biotech, sc-631), JunD (Santa Cruz Biotech sc-74), and Myc (9E10, Vanderbilt Molecular Biology Core, Vanderbilt Antibody and Protein Resource, VAPR, Nashville, TN, 37232, USA). Secondary antibodies were all purchased from Santa Cruz Biotech.

### 4.3. Bioinformatics

#### 4.3.1. UCSC Xena Platform for MAGEB2 Expression Profile

GTEx and TCGA PAN-Cancer expression datasets for *MAGEB2* gene were downloaded. Heart tissue was used as a representative of normal somatic tissue. Of the TCGA Pan-Cancer dataset, only those cancer tissue samples in which *MAGEB2* mRNA levels were greater than two-fold in expression were represented in the histogram.

#### 4.3.2. UCSC Genome Browser for Methylome Profile

Using existing methylome sequencing data published in the UCSC genome browser, we analyzed and counted the methylated CpG sites 1000 bp upstream of *MAGEB2* gene in each of the respective studies. The studies used for heart, testis, and sperm are referenced herein [20]. Ref. [23] was used for the normal, adenomatous polyp, and colon cancer data, and Refs. [24,25] were used for the HCT116 versus HCT116 with double knock-out for DNA methyltransferases (DNMT1 and DNMT3b).

#### 4.3.3. Transcription Factor Analysis

To identify which possible transcription factors may be implicated in regulating *MAGEB2* expression, we interrogated the UCSC genome browser and used the ENCODE Analysis track hub to identify transcription factors that had the highest peaks within the promoter region of *MAGEB2* in cell lines expressing *MAGEB2*. Further analysis using the Eukaryotic Promoter Database indicated the specific locations of the putative binding sites of identified candidates.

### 4.4. Azacytidine Assay

Indicated cells were maintained in media containing DMSO control or 100 nM 5-azacytidine (Sigma Aldrich, A2385) (St. Louis, MO, USA) for one week, at which point cells were harvested and expression of *MAGEB2* and *MAGEL2* mRNA was determined by RT-qPCR.

### 4.5. RT-qPCR

RNA from indicated cell lines and treatment conditions was purified using RNA-easy kit (Qiagen, 74104) (Germantown, MD, USA) using standard manufacturer’s protocol. Following DNAse (Life Technologies, 18068015) (Carlsbad, CA, USA) treatment, 300 ng of RNA from every experimental condition was used for a one-step reverse-transcription and quantitative PCR using a BioRad CFX Maestro thermocycler iQ Sybr Green Master mix (Biorad, 1708880) (Hercules, CA, USA). Pre-designed primers were ordered from Origene. Primer sequences for all genes are given in Table 1. 

### 4.6. Chromatin Immuno-Precipitation

For transcription factor binding studies, chromatin immuno-precipitation (ChIP) and subsequent qPCR was performed using HCT116 and DLD-1 cells. Primers were designed to target the region 250 base pairs upstream of the TSS of *MAGEB2*, a region observed to have consensus binding motifs for all candidate transcription factors. Chromatin immuno-precipitation assays were carried out using the Millipore Magna ChIP^TM^ kit A/G (catalog #17-10085) (Millipore, Burlington, MA, USA). A list of siRNAs and sequences is given in Table 1.

### 4.7. Transient Expression of MAGEB2 in HEK Cells

pCMV-Myc or pCMV-Myc-MAGEB2 plasmids were transfected into HEK cells with Lipofectamine-2000 (Thermo Fisher-11668019) (Waltham, MA, USA) according to manufacturer’s instructions. Cells were harvested at the end of 72 h and lysed for processing for Western blotting or RNA extraction, and samples were processed for RT-qPCR.

### 4.8. Lentiviral (Stable) Expression of MAGE Proteins in HEK Cells

Lentivirus-encoding vector control or *MAGEB2* gene was generated by transfecting 1 µg of pLenti6-blast plasmid encoding myc-tagged MAGE gene along with 0.5 µg of pSPAX and pMD2.G plasmids (Addgene) (Watertown, MA, USA) into 293 FT cells. Both a 48 h and 72 h supernatant containing the lentiviruses were combined, aliquoted, and stored at −80 °C. The viral supernatants were then added to HEK cells along with polybrene (Sigma Aldrich, TR1003-G) (St. Louis, MO, USA) and, 48 h later, cells were moved to media containing blasticidin. Expression of MAGEB2 was tested both by RT-qPCR and Western blotting.

### 4.9. Phenotypic Assays

#### 4.9.1. Population Doubling

In total, 5000 control cells or MAGE-expressing cells were plated in replicates in 6-well dishes and counted using a hemocytometer and automated cell counter every 24 h to determine population doubling times for a period of 9 days. Error bars indicate mean and standard deviation from n = 3 measurements.

#### 4.9.2. Anchorage-Independent Growth

A 0.5% agar (Difco^TM^ Noble Agar from BD Biosciences) (Franklin Lakes, NJ, USA) base layer was made in each well of a 6-well dish by dissolving 1 gm of agar in autoclaved dH_2_O and then mixing the heated agar solution 1:1 with serum-free DMEM. After this layer had solidified, a 0.375% agar cell layer was made by dissolving 0.75 gm of agar in autoclaved dH_2_O and then mixing the heated agar solution 1:1 with serum-free DMEM. In total, 100,000 control or MAGE-expressing cells that were trypsinized to ensure single-cell suspensions were added to this agar solution, and this mixture was plated on top of the base layer. Regular media, or media containing TSP-1 (1 mM), TGFb1 (1 mM), with or without 100 mM SLLK, or LSKL peptide were added on top, and media were changed every two days. Colonies > 100 μm were counted at day 15 using a light microscope. Error bars indicate mean and standard deviation from n = 3 measurements.

### 4.10. RT-qPCR Array

We created a custom-designed Bio-Rad Prime-PCR array based on their cancer collection (Cancer Tier 1 H96 #10025137). We grew 5 × 10 cm dishes of vector control cells, MAGEB2-expressing cells, and MAGEA12-expressing cells to 70% confluence. We harvested cells and prepared RNA as described above. We then performed a 2-step RT-qPCR according to manufacturers’ instruction and analyzed the data and represented fold change in the respective genes in MAGEB2 or MAGEA12-expressing cells over control cells.

### 4.11. ELISA Measurement of TSP-1, TGFβ1, and EREG

Control or MAGEB2-expressing and shControl or shMAGEB2 cells were grown in 10 cm dishes and never allowed to exceed 70% confluence for 2 weeks, and conditioned media were collected every 3 days, pooled, and concentrated using spin concentrators. Supernatants were frozen and used for ELISA assays (TGFβ1 Thermo Fisher: BMS249-4, TSP-1 Thermo Fisher: EHTHBS1, EREG RND Systems: DY1195-05) according to manufacturers’ instructions.

## 5. Conclusions

Our data show that MAGEB2 is a true cancer-testis antigen (Figure 1) and is expressed by the epigenetic regulation of its promoter region, specifically, CpG methylation (Figure 2A,B). The analysis of the consensus transcription factor binding sites within the *MAGEB2* promoter region revealed that four putative transcription factors, ELF1, CTCF, GABPa, or JunD, could regulate *MAGEB2* expression. However, only JunD was enriched on the MAGEB2 promoter, specifically, in cells expressing *MAGEB2* (HCT116) (Figure 2D,E). Furthermore, the knockdown of JunD resulted in a decrease in MAGEB2 expression (Figure 2F). We determined that, when expressed aberrantly, MAGEB2 is both sufficient and necessary to drive cell proliferation (Figure 3). To determine which signaling pathways are altered upon expressing MAGEB2, we performed an RT-qPCR array for cancer-associated genes. We discovered that the expression of MAGEB2 resulted in dampening in the levels of those genes that would typically be up-regulated in response to TGFβ1 (GSEA, Broad Institute) (Figure 4 and Figure 5). Our data indicate that MAGEB2 expression results in a decrease in the levels of TSP-1 mRNA and protein, and, therefore, the levels of secreted TSP-1 and TGFβ1 (Figure 6). When the MAGEB2-induced suppression of the TSP-1/TGFβ1 signaling axis is relieved by either the addition of TSP-1 or TGFβ1, then MAGEB2-expressing cells are no longer pro-proliferative (Figure 7).

## Figures and Tables

**Table 1:** RT qPCR primers.

Gene	Forward Primer	Reverse Primer
*MAGEB2*	CAGCCAGGGGTGAATTCTCAG	TTCTCACGGGCACGGAGCTTA
*MAGEL2*	CACCTTCCTGATGGCTACAGCA	CTGTCCTCTTGGGCTTCCAGAT
*RPLP0* (housekeeping gene)	TCTACAACCCTGAAGTGCTTGAT	CAATCTGCAGACAGACACTGG
*ELF-1* (ChIP)	CTGCTGAGGCACTCCTCAAT	CCATGTCATCTTCAGGTGAACTA
*JUND* (ChIP)	CAGCGAGGAGCAGGAGTT	GAGCTGGTTCTGCTTGTGTAAAT
*GAPB-A* (ChIP)	GGACGGGTCTAGGTGAGACA	TGGCTGGAGTATTTCAAAGGAT
*CTCF* (ChIP)	AAGAAAGATGCGCTCTAAGAAAGA	CATCCTCATTGTCGTCCAGA
*JunD*	ATCGACATGGACACGCAGGAGC	CTCCGTGTTCTGACTCTTGAGG
*KDR*	GGAACCTCACTATCCGCAGAGT	CCAAGTTCGTCTTTTCCTGGGC
*TGFb1*	TACCTGAACCCGTGTTGCTCTC	GTTGCTGAGGTATCGCCAGGAA
*CTNNB1*	CACAAGCAGAGTGCTGAAGGTG	GATTCCTGAGAGTCCAAAGACAG
*CD44*	CCAGAAGGAACAGTGGTTTGGC	ACTGTCCTCTGGGCTTGGTGTT
*MUC1*	CCTACCATCCTATGAGCGAGTAC	GCTGGGTTTGTGTAAGAGAGGC
*APC*	AGGCTGCATGAGAGCACTTGTG	CACACTTCCAACTTCTCGCAACG
*CDH1*	GCCTCCTGAAAAGAGAGTGGAAG	TGGCAGTGTCTCTCCAAATCCG
*EREG*	CTTATCACAGTCGTCGGTTCCAC	GCCATTCAGACTTGCGGCAACT
*SMAD4*	CTACCAGCACTGCCAACTTTCC	CCTGATGCTATCTGCAACAGTCC
*THBS1*	GCTGGAAATGTGGTGCTTGTCC	CTCCATTGTGGTTGAAGCAGGC

## Data Availability

All data generated are included in this manuscript. All datasets analyzed are available at https://genome.ucsc.edu/cgi-bin/hgGateway, accessed on 2 February 2019 and https://xenabrowser.net, accessed on 2 February 2019 and the studies are referenced in this manuscript [16,21,22,23,24,25].
